# Craig Driver & Ross Warren (architects) will present examples of an innovative waymaking (wayfinding) concept from a current development in Norwich, UK

**DOI:** 10.1192/j.eurpsy.2023.174

**Published:** 2023-07-19

**Authors:** R. D. Warren

**Affiliations:** Architecture, ARB, London, United Kingdom

## Abstract

**Abstract:**

A new interpretation of the normalised “Wayfinding” design task offers the opportunity to become an important element of the larger clinical and architectural project for a new “core” expansion of a large regional psychiatric hospital in the South-East of the UK. We call this new approach *Waymaking*, as it goes beyond signage, leveraging our deep-set knowledge and understanding of the entire project at all scales.

Waymaking at the **Rivers Centre for Mental Health** (Rivers) begins with the exploration of movement narratives into and around its larger site. It turns a classic design task into a design opportunity on all scales, starting with an urban design and planning perspective, through to the architectural and landscape design decisions outside of the building and into the specific on-ward atmospheres in a manner integrated with the detail interior design decisions of colour, built-in-furniture and others.

Rivers has been carefully composed out of existing structures as well as smaller new-build and extension buildings. These are all set within a large, sloping site of noteworthy natural beauty. As such, Rivers can well be understood as a hillside village or campus of health - rather than as a traditional “hospital.” As a health village, Rivers provides spatial sequencing as the landscape design directly introduces a series of smaller, more human scale spaces built and natural all of which together aid in orientation and identity across the site. This will help support the daily use of the buildings by all stakeholders.

This strategy has been “baked-in“ to the architectural design as well, strategically distributed retreat/recovery spaces allow for space for de-escalation or relaxation. These can be found in the form of regular niches in the hallways and “porch” entrance spaces, usually with built-in benches and bespoke lighting elements. In addition to creating orientation affordances, these also provide opportunities for neurodivergent persons (ie. ASD, learning disabilities, etc.) to better understand and master independent movement around the Centre.

**Disclosure of Interest:**

None Declared

